# Perirenal fat as a potential marker and therapeutic target for metabolic syndrome: insights from a multicenter randomized controlled trial

**DOI:** 10.3389/fendo.2025.1557701

**Published:** 2025-05-23

**Authors:** Yang Hua, Meng-Huan Li, Yu-Xuan Lou, Ke-Rui Zhang, Jia-Ming Yang, Yan-Hui Sheng, Yu-Qing Zhang, Chuan-li Cheng, Chao Zou, Ting-ting Wu, Xiang-Qing Kong, Wei Sun

**Affiliations:** ^1^ Department of Cardiology, The First Affiliated Hospital with Nanjing Medical University, Nanjing, China; ^2^ Department of Cardiology, The First Affiliated Hospital with Nanchang Medical University, Nanchang, China; ^3^ Cardiovascular Research Center, The Affiliated Suzhou Hospital with Nanjing Medical University, Gusu School, Suzhou, China; ^4^ Department of Cardiology, The Affiliated Jiangning Hospital with Nanjing Medical University, Nanjing, China; ^5^ Paul C. Lauterbur Research Center for Biomedical Imaging, Shenzhen Institutes of Advanced Technology, Chinese Academy of Sciences, Shenzhen, China

**Keywords:** metabolic syndrome, perirenal fat, ultrasonography, visceral fat, Chinese population

## Abstract

**Background:**

Metabolic syndrome (MetS) represents a constellation of metabolic abnormalities. Perirenal fat is a type of visceral fat surrounding the kidneys and possesses distinct anatomical and physiological features. This study aims to investigate the association between perirenal fat volume (PrFV) and MetS in Chinese adults.

**Methods:**

We conducted a *post-hoc* cross-sectional analysis within a multicenter, randomized clinical trial. Demographic information, anthropometric data and laboratory tests were obtained from the electronic data capture system. PrFV was assessed and measured by ultrasonography. Subcutaneous and visceral fat volume were quantified by abdominal MRI. Individuals were categorized according to PrFV tertiles, and Spearman correlation analysis was performed to investigate the correlation between PrFV and metabolic profiles. Adjusted multivariable regression models were employed to investigate the relationship of PrFV with MetS. The receiver operating characteristic curve was used to identify the value of PrFV for predicting MetS.

**Results:**

Among 100 enrolled subjects, the median age was 50.0 (40.0-60.0) years, and 75% were male. Spearman correlation analysis revealed significant positive correlations between PrFV and total cholesterol (r = 0.24, *P* = 0.02), triglycerides (r = 0.32, *P* = 0.001), LDL-C (r = 0.21, *P* = 0.04), diastolic blood pressure (r = 0.24, *P* = 0.02), BMI (r = 0.39, *P* < 0.001), waist circumference (r = 0.39, *P* < 0.001), and uric acid (r = 0.40, *P* < 0.001). In the fully-adjusted multivariable regression model, individuals in the highest tertile of PrFV exhibited a higher risk of MetS (Odds ratio = 4.48, 95% Confidence interval: 1.25-17.6). The area under the curve (AUC) of PrFV for predicting MetS was higher than subcutaneous and visceral fat volume.

**Conclusion:**

Increased PrFV was positively associated with a higher risk of MetS in Chinese adults. Perirenal fat may serve as a surrogate marker and potential therapeutic target for MetS.

**Clinical trial registration:**

https://clinicaltrials.gov/, identifier NCT 05049096.

## 1 Introduction

Metabolic syndrome (MetS), though subtly varied in definition, broadly encompasses a cluster of pathological conditions characterized by abdominal obesity, insulin resistance, hypertension, and hyperlipidemia. Factors such as excessive calorie intake over physical expenditure, sedentary lifestyle habits, low quality of diet species, and genetic/epigenetic background contribute to the development of MetS (1). With the high-speed socio-economy progress and the widespread adoption of Western lifestyles, MetS is rapidly sweeping developing countries, particularly in urban areas. Notably, the prevalence of MetS in China stands at 31.1%, with an estimated 450 million individuals affected (2). This figure compares to 37.1% in the US (3) and 10.5% in the Europe (4), highlighting the significant burden that MetS poses a long-term and far-reaching health risk for cardiovascular and cerebrovascular diseases in China.

Given the critical role of body fat distribution in the context of MetS, it is essential to examine the specific contributions of different types of adipose tissue. Visceral adipose tissue (VAT) is distributed on and adjacent to the surface of abdominal organs within the mesentery and omentum. Compared to subcutaneous adipose tissue (SAT), VAT is more cellular, vascular, and active in metabolism and secretion function (5). Consequently, VAT is intricately tied to an elevated risk of cardiovascular diseases, metabolic parameters, and cardiovascular mortality. Numerous studies have firmly established a robust correlation between excess VAT accumulation and a range of metabolic disorders, including dyslipidemia, hypertension, hyperglycemia, etc (6–8).

Among VAT, a significant type of adipose tissue that warrants attention is perirenal fat, which may have unique implications for metabolic health. Perirenal fat, also known as perirenal adipose tissue (PRAT), is a layer of fat surrounding the kidney and lies within the renal fascia. PRAT shares typical characteristics with VAT, including its morphological proximity to abdominal organs, robust vascularization and innervation, and susceptibility to lipolysis. A recent study reported that PRAT ablation was associated with reduced pathological high blood pressure (without affecting normal blood pressure) (9). In this context, PRAT may distinguish itself from other types of VAT, as it not only serves as a passive energy storage organ but also actively participates in specific biological functions, such as sensory neuron activity regulation, adipokines secretion, and fat-kidney interaction (10).

Given its anatomical location and unique biological characteristics, PRAT may provide valuable insights into the relationship between adipose tissue and metabolic syndrome. Owing to the anatomical renal location and morphological fat distribution (retroperitoneal organ with a relatively fixed position), the inferior PRAT accumulates the most abundant fat within the kidney fascia and is minimally affected by factors such as body size, gastrointestinal peristalsis, and breathing. Therefore, the inferior PRAT serves as a straightforward window for visceral fat assessment. Ultrasonography offers a convenient and nonradiative alternative to computed tomography (CT) or magnetic resonance imaging (MRI). Studies have demonstrated that inferior perirenal fat measured by ultrasonography is highly correlated with visceral fat content measured by MRI (10) and exhibits comparable accuracy to that measured by CT (11). In this cross-sectional study, we utilized ultrasonography to measure inferior PRAT and employed MRI to quantify SAT and VAT. We aimed to explore the relationship between PRAT and MetS among Chinese adults.

## 2 Materials and methods

### 2.1 Participants

The participants were derived from a multicenter, randomized, controlled clinical trial aimed at investigating the efficacy of focused power ultrasound inferior PRAT modification therapy in reducing blood pressure (PARADISE-HTN, NCT05049096). Between December 2021 and June 2023, one hundred adult subjects were consecutively enrolled from three centers. Ultimately, our study comprised 35 subjects from the First Affiliated Hospital of Nanjing Medical University, 43 from the Affiliated Suzhou Hospital of Nanjing Medical University, and 22 from the Affiliated Jiangning Hospital of Nanjing Medical University.

Subjects were meticulously interviewed for demographic information and medical history. The main exclusion criteria were rigorously defined and as follows: secondary hypertension, history of kidney or kidney surrounding tissue surgery/infection, complicated with severe heart disease (new myocardial infarction in the last six weeks, malignant heart rhythm, severe valvular heart disease, etc.), renal/liver impairment (alanine aminotransferase, aspartate aminotransferase or creatinine greater than two times of the upper limit of standard reference), urinary calculi or hematuria, type I diabetes or uncontrolled type II diabetes (Glycated Hemoglobin A1C (HBA1C) > 7.0%). This study was approved by the Institutional Review Boards of all participating branch centers, and informed consent was obtained from all enrolled participants.

### 2.2 Anthropometric measurements

Office blood pressure (systolic blood pressure, SBP; diastolic blood pressure, DBP) was measured consecutively three times in a seated position and averaged with an interval of 1–2 minutes after 5 minutes of rest (OMRON, HBP-9030). Body mass index (BMI) was calculated by dividing weight by the square of their height (kg/m^2^). Waist circumstance (WC) was measured as the length of a tape measure around the abdomen at the level of the midpoint between the upper margin of the iliac bone and the lower margin of the costal bone. Socio-demographic information (including age, gender, marital status, education level) and medical history (including comorbidities, medication use) were obtained by self-report, whereas alcohol consumption and smoking history were collected by dietary questionnaire.

### 2.3 Blood sampling and analysis

Fasting morning blood samples of subjects were collected and tested by a third-party laboratory (Kingmed Diagnostics, Nanjing). Serum creatinine (SCr), serum uric acid (SUA), low-density lipoprotein cholesterol (LDL), high-density lipoprotein cholesterol (HDL), total cholesterol (TC), triglycerides (TG), and fast plasma glucose (FPG) were measured by standard enzymatic methods (Au5800, Beckman, United States). HBA1C was measured by high-performance liquid chromatography methods (G11-90SL, TOSOH, Japan). Triglyceride-glucose index (TyG) is calculated using the formula: TyG = ln (TG (mg/dL) × FPG (mg/dL)).

### 2.4 Ultrasonic measurement

**Figure 1:**
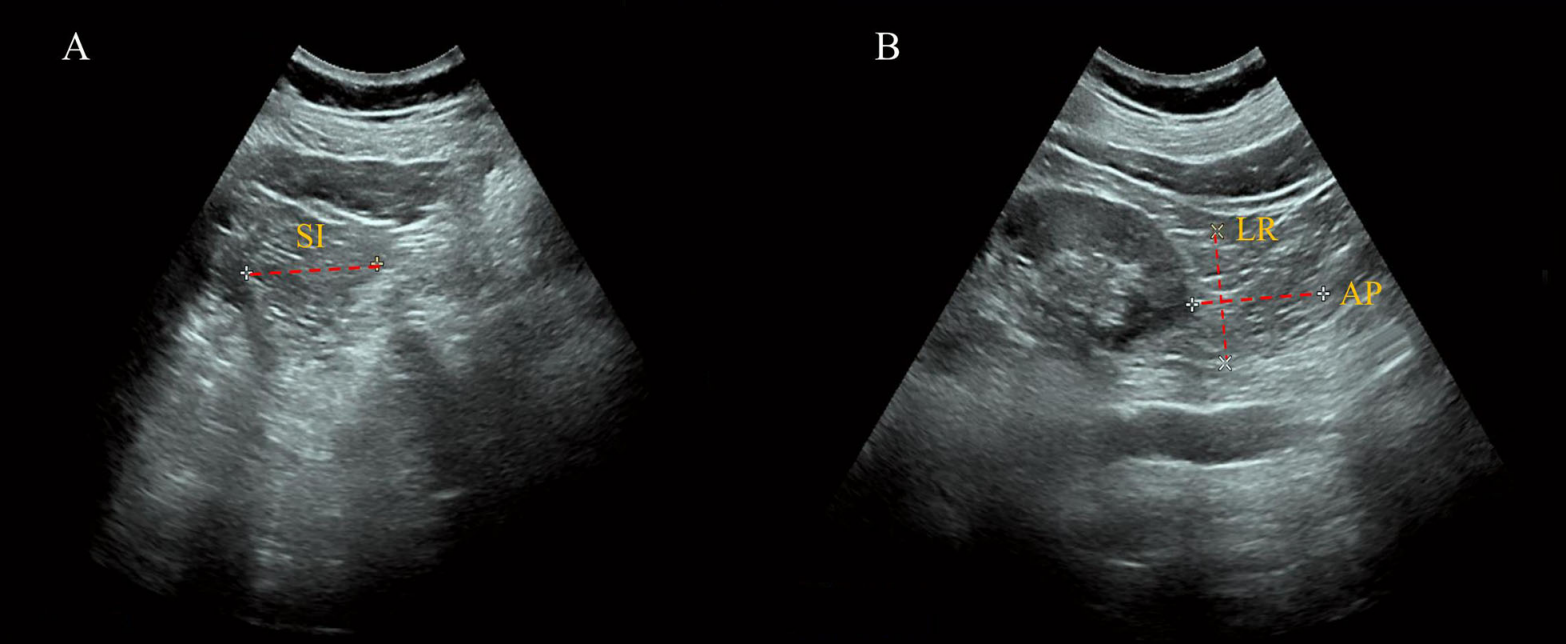
Measurement of perirenal fat volume by ultrasonography. **(A)** Long-Axis View: The superior-inferior (SI) diameter of the inferior PRAT is measured, defined as the maximal thickness between the fibrous membrane and the renal fascia. **(B)** Short-Axis View: The left-right (LR) diameter and anterior-posterior (AP) diameter of the inferior PRAT are measured, illustrating the three-dimensional assessment of perirenal fat volume.

In our study, a qualified ultrasound physician measured inferior PRAT in three sections: longitudinally, transversally, and anterior-posteriorly (Philips Ultrasound, Bothell, WA, USA). The Subjects were positioned on their side on the examination bed with their waist skin exposed. With the ultrasound probe aligned parallel to the body’s long axis, the low echo area close to the inferior pole of the kidney was identified as the inferior PRAT. In this long-axis section, the maximal superior-inferior (SI) diameter of the inferior PRAT was measured. The short-axis section of the kidney was obtained by rotating the probe vertically to the skin and moving slowly downward. The low echo mass that moved up and down slightly with respiration was the inferior PRAT. At the point where the inferior kidney disappeared from view, the maximum left-right (LR) diameter and anterior-posterior (AP) diameter were instantly measured (**Figure 1**). The perirenal fat volume (PrFV) was averaged by multiplying the two sides’ SI, LR, and AP diameters.

### 2.5 Measurement of subcutaneous and visceral fat volume

MRI for all participants were performed using a 3.0 T MRI clinical scanner (uMR770, United Imaging Healthcare in The First Affiliated hospital of Nanjing Medical University, Siemens Prisma in The Affiliated Jiangning Hospital of Nanjing Medical University, Siemens Skyra in The Affiliated Suzhou Hospital of Nanjing Medical University). For proton density fat fraction (PDFF) imaging, a six-echo gradient echo imaging sequence was employed for image acquisition with the following specific parameters: repetition time (TR) = 10 ms, echo time (TE) = 1.55/2.99/4.43/5.87/7.31/8.75 ms, bandwidth = 1000 Hz/pixel, flip angle = 3°, field of view (FOV) = 300 × 400 mm, imaging resolution = 144 × 192, slice thickness = 4 mm (uMR770); TR = 9 ms, TE = 1.14/2.46/3.69/4.92/6.15/7.38 ms, bandwidth = 1085 Hz/pixel, flip angle = 3°, FOV = 350 × 400 mm, imaging resolution = 168 × 192, slice thickness= 3 mm (Siemens Prisma and Siemens Skyra).

A deep learning-based tool for automatic whole-body adipose tissue segmentation previously reported was employed (12). This network is capable of automatically segmenting the input PDFF images of various body parts, distinguishing total adipose tissue (TAT) into SAT and VAT, where VAT is defined as total adipose tissue excluding SAT: VAT = TAT - SAT. The automatic image segmentation was conducted in Python 3.9. Based on the results of the aforementioned automatic segmentation, the adipose tissue volume of each volunteer was calculated, including the subcutaneous fat volume (SFV) and visceral fat volume (VFV).

### 2.6 Definition of MetS

According to Diabetology Branch of Chinese Medical Association 2020 Diagnostic criteria of Chinese Guidelines for the Prevention and Treatment of Type 2 diabetes (13), MetS was defined as the presence of any three of the following metabolic abnormalities: (1) abdominal obesity (WC ≥ 90 cm for male, or ≥ 85 cm for female; (2) hyperglycemia: FPG ≥ 6.1 mmol/L, a 2-hour postprandial blood glucose level of ≥ 7.8 mmol/L, a prior diagnosis of diabetes, or the use of hypoglycemic medication; (3) elevated blood pressure: SBP ≥ 130 mmHg or DBP ≥ 85 mmHg, or diagnosed as hypertension or receive anti-hypertensive medication; (4) fasting TG ≥ 1.7 mmol/L; (5) fasting HDL-C < 1.04 mmol/L. As we did not measure 2-hour postprandial blood glucose for participants, we employed a more stable index, HBA1C, to assess glucose metabolism, with a cut-off of ≥ 6.5% for hyperglycemia.

### 2.7 Statistical analysis

The study population were stratified into three groups based on average PrFV tertiles, with low tertile set as the reference (Tertile 1, 29.8 cm^3^; Tertile 2, 54.9 cm^3^; Tertile 3, 101.6 cm^3^). Continuous variables with a normal distribution were represented as mean ± standard deviation, whereas continuous variables with skewed distribution and categorical variables were described as median with interquartile range and percentages, respectively. Baseline characteristics between the three groups were compared using the chi-square test (categorical variables), one-way ANOVA test (normal distribution), or Kruskal-Wallis test (skewed distribution). Spearman correlation analysis was performed to investigate any simple correlation between PrFV and other MetS component variables. Moreover, the enrolled population were also categorized according to the number of MetS components. Non-parametric Kruskal-Wallis tests were used to discern any relation between PrFV and the number of MetS components.

Three adjusted multivariate regression models with odds ratios (ORs) and corresponding 95% confidence intervals (CIs) were used to explore the associations between PrFV (either as continuous variable or as categorical PrFV tertiles) and MetS: Model 1 did not include any adjustments; Model 2 was adjusted for age and gender; Model 3 was further adjusted for smoking status, drinking status, BMI, subcutaneous fat volume and visceral fat volume. The receiver operating characteristic curves were used to compare the identifying value of MetS between PrFV and SFV and VFV. Additionally, supplementary analysis was conducted using adjusted multivariate regression models to explore the association between PrFV and typical metabolic disorders, such as hyperlipidemia, hyperglycemia, and hyperuricemia. All statistical analysis was performed via R software version 3.6.1. P-value < 0.05 was considered statistically significant for all analyses.

## 3 Results

### 3.1 Baseline characteristics of the study population

The baseline characters of the study population stratified by PrFV tertiles are shown in **Table 1**. Among the general population of 100 enrolled subjects, the median age was 50.0 (40.0-60.0) years, and 75% were males. There were significant differences in gender, BMI, WC, CRP, TG, TyG, SUA, among the three groups. Subjects in the highest PrFV tertile were more likely to be male (*P* < 0.05) and had higher BMI and WC (*P* < 0.01). However, other demographic information, such as age, smoking and drinking status, did not differ among the groups. Compared to the lowest PrFV tertile, higher PrFV tertile was associated with higher levels of CRP, TG, TyG, and SUA (all *P* < 0.05). Despite an increasing trend of Scr, LDL-C, FPG, and DBP along with higher PrFV, no significant statistical differences were observed among the groups.

**Table 1:** Characteristics of the study population stratified across average PrFV tertiles.

	Total (n=100)	Tertile 1 (n=33)	Tertile 2 (n=33)	Tertile 3 (n=34)	*P*
Age, year	50.0 (40.0-60.0)	51.0 (38.0-62.0)	53.0 (40.5-60.5)	46.5 (40.0-53.5)	0.403
Male (%)	75 (75.0)	20 (60.6)	25 (75.8)	30 (88.2)	0.033
BMI, kg/m^2^	27.3 (24.8-30.3)	25.2 (23,2-28.7)	27.3 (24.3-30.0)	29.1 (26.3-31.1)	0.008
WC, cm	95.0 (87.6-102)	92.0 (85.0-98.8)	95.0 (86.0-102.0)	101.0 (92.0-105.0)	0.009
Smoker (%)	27 (27.0)	5 (15.2)	9 (27.3)	13 (38.2)	0.104
Drinker (%)	30 (30.0)	7 (21.2)	9 (27.3)	14 (41.2)	0.187
CRP, mmol/L	1.10 (0.65-2.29)	0.87 (0.54-1.95)	1.04 (0.65-1.83)	1.66 (0.75-3.23)	0.04
SCr, mmol/L	74.0 (16.4)	70.4 (16.8)	73.2 (15.4)	78.3 (16.5)	0.134
TC, mmol/L	4.9 (1.2)	4.6 (1.2)	4.7 (1.0)	5.2 (1.1)	0.078
TG, mmol/L	1.53 (1.14-2.47)	1.32 (0.96-1.65)	1.40 (1.08-1.91)	2.52 (1.29-3.71)	0.002
LDL-C, mmol/L	3.05 (0.73)	2.87 (0.73)	3.12 (0.62)	3.15 (0.80)	0.234
HDL-C, mmol/L	1.09 (0.95-1.25)	1.13 (1.02-1.22)	1.01 (0.92-1.23)	1.10 (0.89-1.31)	0.396
FPG, mmol/L	5.36 (4.83-6.21)	5.16 (4.52-5.94)	5.44 (4.89-6.17)	5.47 (4.97-6.63)	0.180
SUA, mmol/L	363.4 (94.2)	321.6 (80.7)	351.9 (82.7)	415.0 (95.2)	<0.001
TyG	8.80 (8.40-9.30)	8.50 (8.25-9.05)	8.70 (8.40-9.10)	9.30 (8.68-9.75)	0.001
SBP, mmHg	151.1 (8.7)	151.3 (9.0)	150.2 (8.7)	151.9 (8.6)	0.717
DBP, mmHg	89.9 (10.8)	87.4 (12.1)	89.8 (9.6)	92.5 (10.2)	0.147
Subcutaneous fat volume, cm^3^	1510.2(653.7)	1531.2(754.3)	1459.0(618.4)	1539.4(600.5)	0.811
Visceral fat volume, cm^3^	2069.3(775.5)	1847.8(867.1)	2049.0(551.8)	2302.9(824.4)	0.043
Average PrFV, cm^3^	55.6 (37.0-92.7)	29.8 (23.6-37.8)	54.9 (49.5-66.5)	101.6 (92.4-114.1)	<0.001

Data are presented as percentages for categorical variables, median (interquartile range) for continuous variables with skewed distribution and mean (standardized diﬀerences) with normal distribution.

PrFV, perirenal fat volume; BMI, body mass index; WC, waist circumference; CRP, C-reactive protein; SCr, serum creatinine; TC, total cholesterol; TG, triglycerides; LDL-C, low density lipoprotein-cholesterol; HDL-C, high density lipoprotein-cholesterol; FPG, fast plasma glucose; SUA, serum uric acid; TyG, triglyceride-glucose index; SBP, systolic blood pressure; DBP, diastolic blood pressure.

### 3.2 Measurement of SFV and VFV

**Figure 2:**
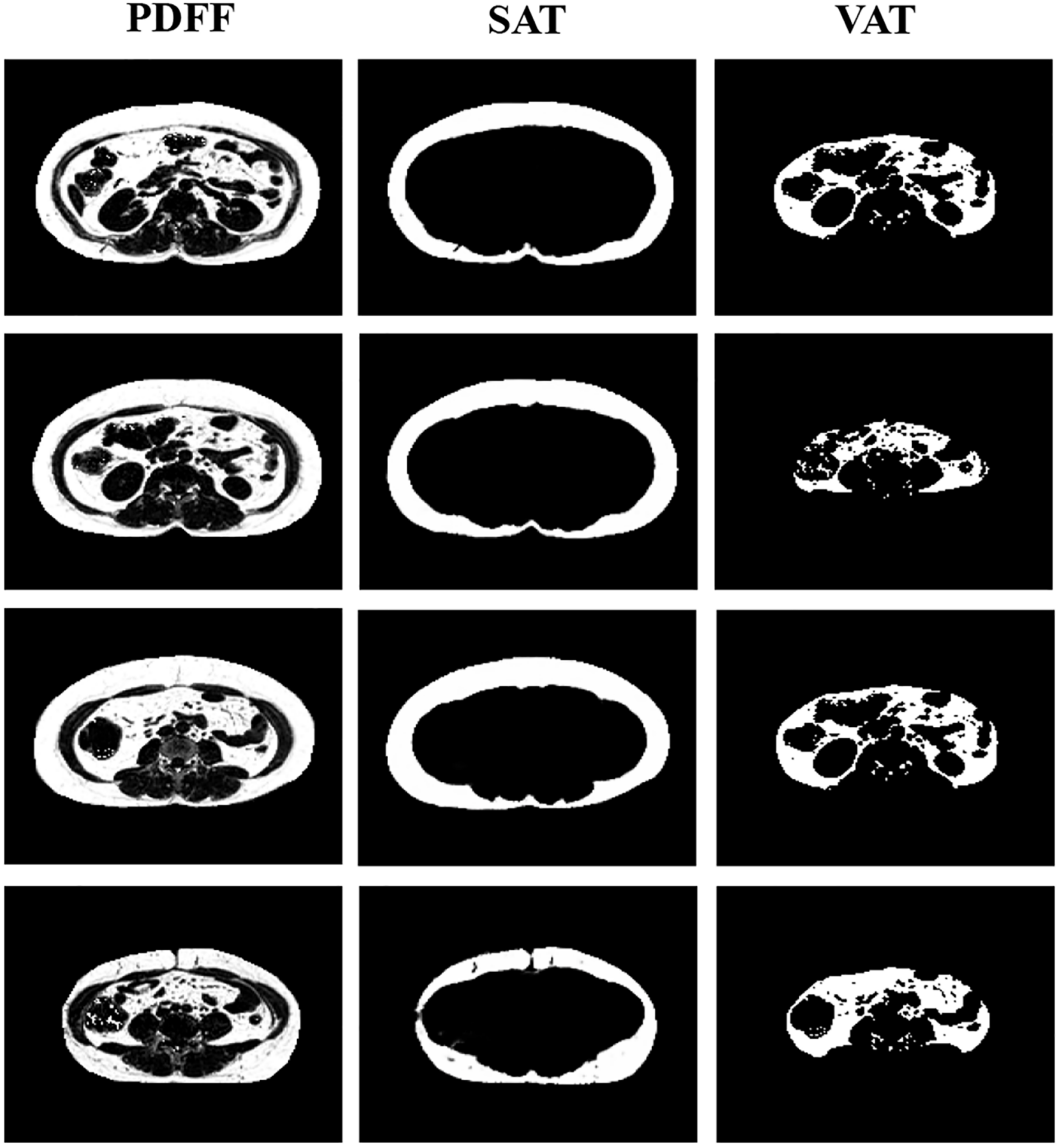
Proton density fat fraction (PDFF) imaging of the abdominal region. The segmentation distinguishes between subcutaneous adipose tissue (SAT) and visceral adipose tissue (VAT), providing a comprehensive view of fat distribution in the abdominal area.

**Figure 2** displays the PDFF images of abdominal area of a female volunteer. TAT in abdominal area was further distinguished into SAT and VAT through automatic segmentation of adipose tissue from magnetic resonance fat fraction images based on machine learning. Each volunteer underwent scanning of the abdominal region, with 24 to 32 slices being acquired. After segmentation of each slice, the cumulative pixel values represent the fat volume of SAT and VAT. SFV and VFV is then calculated by averaging the fat volume across different slices. The average SFV and VFV among populations were 1510.2 ± 653.7 cm^3^ and 2069.3 ± 775.5 cm^3^, respectively (**Table 1**). There was significant positive correlation between increasing PrFV tertiles and VFV (Tertile 1: 1847.8 ± 867.1 cm^3^; Tertile 2: 2049.0 ± 551.8 cm^3^; Tertile 3: 2302.9 ± 824.4 cm^3^, *P* = 0.043). However, no significant statistical differences was observed of SFV among the groups.

### 3.3 Correlations of PrFV and clinical variables of MetS

**Figure 3:**
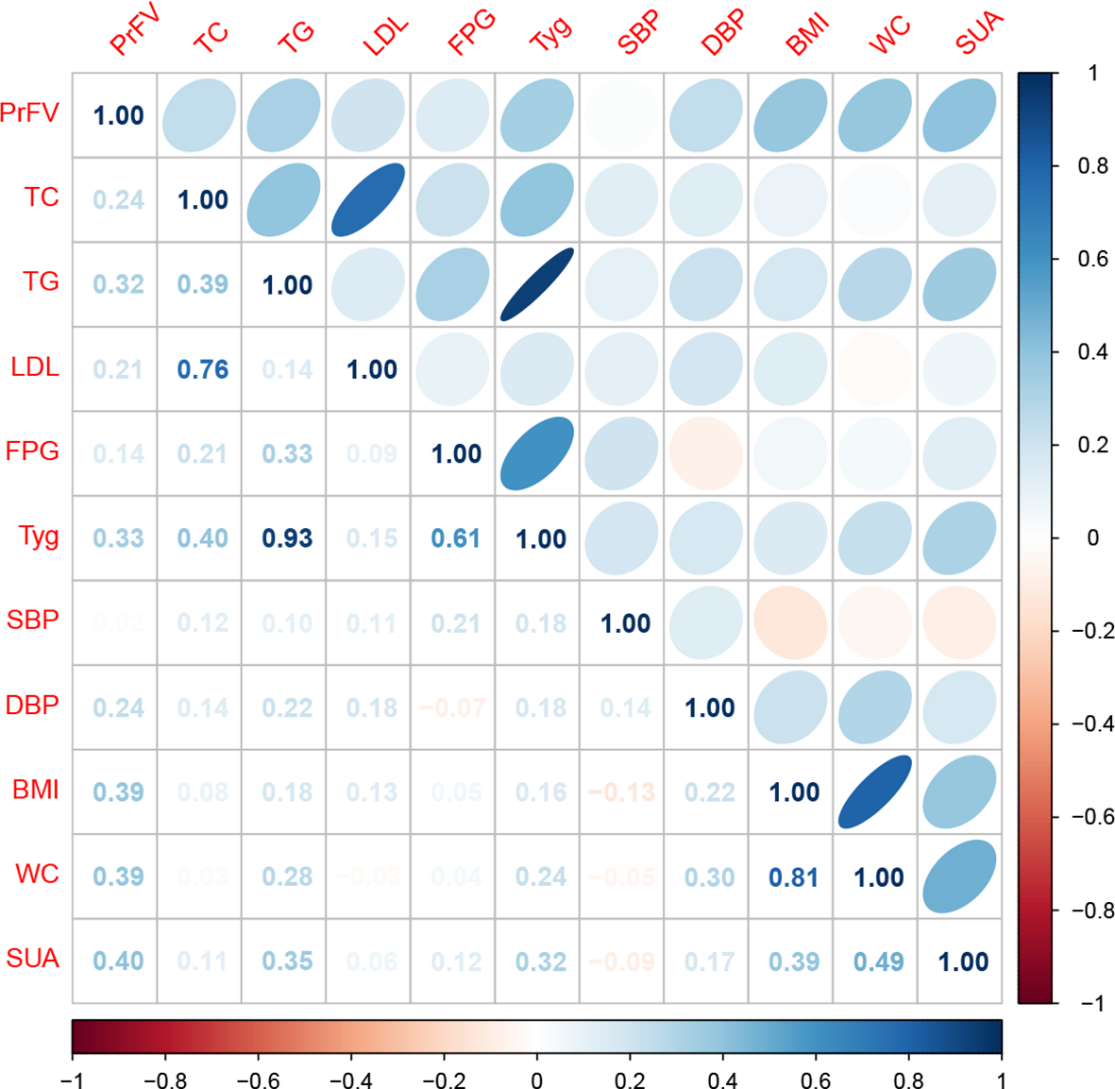
Spearman correlation analysis of the association between perirenal fat volume (PrFV) and metabolic profiles. TC, total cholesterol; TG, triglycerides; LDL-C, low-density lipoprotein cholesterol; HDL-C, high -density lipoprotein cholesterol; FPG, fast plasma glucose; TyG, triglyceride-glucose index; SUA, serum uric acid; SBP, systolic blood pressure; DBP, diastolic blood pressure; BMI, body mass index; WC, waist circumference.

Spearman correlation analysis was employed to assess any direct association between PrFV and component clinical variables of MetS, including TC, TG, LDL-C, HDL-C, FPG, SBP, DBP, BMI, and WC (**Table 2**, **Figure 3**). The results revealed strong and positive correlations between PrFV and TC (r = 0.24, *P* = 0.02), TG (r = 0.32, *P* = 0.001), LDL-C (r = 0.21, *P* = 0.04), DBP (r = 0.24, *P* = 0.02), BMI (r = 0.39, *P* < 0.001), and WC (r = 0.39, *P* < 0.001). Conversely, no significant correlation was found between PrFV and HDL-C (r = -0.07, *P* = 0.48), FPG (r = 0.14, *P* = 0.16), and SBP (r = 0.02, *P* = 0.88). Moreover, we found a robust positive correlation between PrFV and SUA (r = 0.40, *P* < 0.001), a hematological marker of purine metabolism.

**Table 2:** Correlations between PrFV and clinical variables of MetS in the study population.

Variables	Average PrFV, cm^3^	
	r	*P*
TC, mmol/L	0.24	0.02*
TG, mmol/L	0.32	0.001**
LDL-C, mmol/L	0.21	0.04*
HDL-C, mmol/L	-0.07	0.48
FPG, mmol/L	0.14	0.16
SUA, mmol/L	0.40	<0.001***
SBP, mmHg	0.02	0.88
DBP, mmHg	0.24	0.02*
BMI, kg/m^2^	0.39	<0.001***
WC, cm	0.39	<0.001***

TC, total cholesterol; TG, triglycerides; LDL-C, low-density lipoprotein cholesterol; HDL-C, high -density lipoprotein cholesterol; FPG, fast plasma glucose; SUA, serum uric acid; SBP, systolic blood pressure; DBP, diastolic blood pressure; BMI, body mass index; WC, waist circumference.

***P < 0.001, **P < 0.01 and *P < 0.05.

### 3.4 Association between PrFV and MetS

The results of multivariable logistic regression analysis are presented in **Table 3**. When treated as a continuous variable, an increase in PrFV was positively associated with the presence of MetS in all three regression models. When treated as an ordinal categorical variable, individuals in the higher PrFV tertile were associated with an increased probability of MetS. After full adjustment for age, gender, smoking status, drinking status, BMI, SFV and VFV, individuals in the highest PrFV tertile had a significantly higher risk of MetS compared to those in the lowest PrFV tertile. The ORs with 95% CIs for MetS across increasing tertiles were 2.20 (0.67, 7.48) and 4.48 (1.25, 17.6) in the fully adjusted model.

**Table 3:** Logistic regression models between PrFV and MetS.

PrFV	Crude model (Model 1)		Model 2		Model 3	
	Odds ratio (95%CI)	P-value	Odds ratio (95%CI)	P-value	Odds ratio (95%CI)	P-value
PrFV (continiuos variable)	1.03 (1.02-1.05)	<0.001	1.03 (1.02-1.05)	<0.001	1.03 (1.01-1.05)	0.01
PrFV (categorical variable)						
Q1	Ref.		Ref.		Ref.	
Q2	3.06 (1.14-8.60)	0.029	2.77 (1.01-8.01)	0.05	2.20 (0.67-7.48)	0.20
Q3	8.18 (2.77-27.2)	<0.001	7.15 (2.33-24.6)	<0.001	4.48 (1.25-17.6)	0.03

CI, Confidence interval.

Model 1 was adjusted for none. Model 2 was adjusted for age and gender. Model 3 was further adjusted for smoking status, drinking status, BMI, subcutaneous fat volume and visceral fat volume.

The association between PrFV and MetS components is shown in **Table 4**. We categorized the population into five groups according to the number of MetS component items they possessed. Notably, the group with three MetS components comprised the largest subset, consisting of 38 cases. There were statistically significant differences in PrFV across the groups (*P* < 0.001). The median value of PrFV for the five groups were 28.1, 45.4, 74.6, 83.4, and 53.9 cm^3^, respectively. A positive and growing trend was observed between PrFV and the number of MetS component items, except for those individuals with five MetS components.

**Table 4:** Relationship between PrFV and MetS components.

Component items of MS	Number of cases	PrFV (cm^3^)	Mean of rank	P
1	6	28.1 (20.7-35.8)	16.1	<0.001
2	33	45.4 (31.5-59.5)	38.6	
3	33	74.6 (52.1-101.6)	61.8	
4	21	83.4 (43.0-113.7)	62.1	
5	7	53.9 (41.5-82.8)	48.23	

Non-parametric Kruskal-Wallis test was used to compare the median between the groups.

**Supplementary Table S1** illustrates the association between PrFV and typical metabolic disorders, namely hyperlipidemia, hyperglycemia, and hyperuricemia. There was no significant association of PrFV with the prevalence of hyperglycemia. However, when treated as a continuous variable, PrFV was positively and significantly correlated with the prevalence of both hyperlipidemia and hyperuricemia. The ORs with 95% CIs for hyperuricemia across increasing PrFV tertiles were 1.09 (0.28, 4.31) and 5.20 (1.47, 20.45) after full adjustment.

### 3.5 Comparison of PrFV, SFV, VFV in identifying MetS

**Figure 4:**
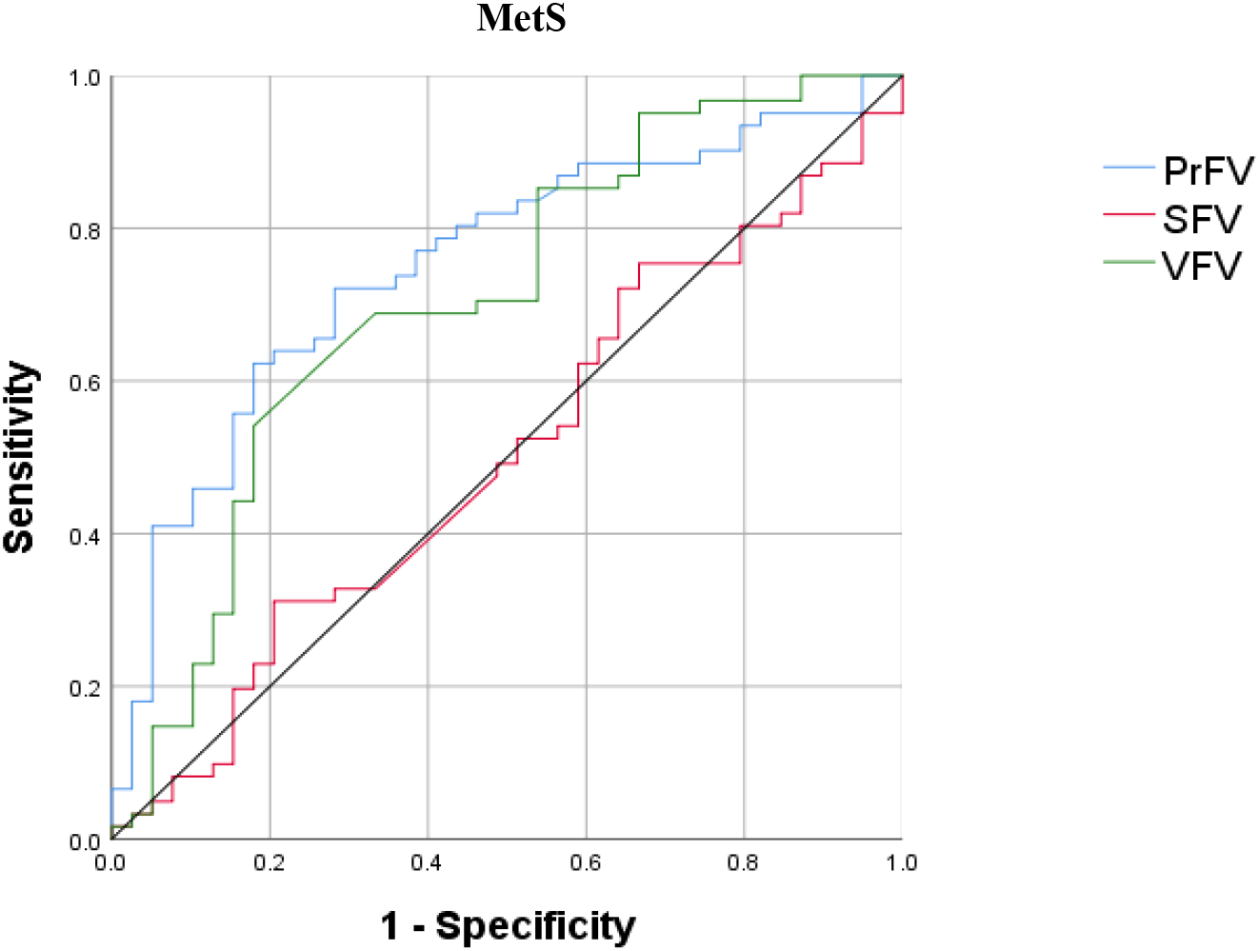
Receiver operating characteristic (ROC) curves for identifying metabolic syndrome (MetS). PrFV, perirenal fat volume; SFV, subcutaneous fat volume; VFV, visceral fat volume.

**Figure 4** shows the performance for evaluating the value of PrFV, SFV, and VFV for identifying MetS. The AUCs (95% CI) of PrFV, SFV, and VFV were 0.753 (0.656-0.849), 0.706 (0.598-0.813), and 0.503 (0.386-0.619), respectively. The AUC of PrFV was higher than SFV (ΔAUC = 0.25, *P* = 0.001) and VFV (ΔAUC = 0.047, *P* = 0.51). The optimal cutoff values of PrFV were 62.5 cm^3^, with a sensitivity of 62.3% and a specificity of 82.1% (**Table 5**).

**Table 5:** ROC curve analysis of PrFV, SFV, and VFV in identifying MetS.

Variables	AUC (95%CI)	Cutoff value	Sensitivity (%)	Specificity (%)
PrFV (cm^3^)	0.753 (0.656–0.849)	62.5	62.3	82.1
SFV (cm^3^)	0.706 (0.598–0.813)	1645.4	31.1	79.5
VFV (cm^3^)	0.503 (0.386–0.619)	2087.6	54.1	82.1

PrFV, perirenal fat volume; SFV, subcutaneous fat volume; VFV, visceral fat volume; MetS, metabolic syndrome.

## 4 Discussion

MetS is a combination of metabolically-related risk factors, including abdominal fat, hypertension, as well as disruptions in lipid and glucose metabolism (14). These factors may independently or synergistically contribute to atherosclerotic heart disease and type 2 diabetes mellitus. Unfortunately, MetS has garnered insufficient attention and understanding among clinicians, particularly in developing nations, where a holistic view of this systemic disorder is frequently absent, resulting in suboptimal medical management strategies. Thus, MetS has emerged as a formidable health threat and a pressing issue in both clinical practice and public health (15). In light of this pressing issue, our cross-sectional study among Chinese adults delved into a distinctive visceral fat depot known as PRAT. We observed a positive relation between PRAT and several component variables of MetS, including TC, TG, LDL-C, DBP, BMI, and WC. Notably, we also uncovered a significant and independent positive link between PRAT and the prevalence of both MetS and hyperuricemia, irrespective of age, gender, smoking habits, alcohol consumption, BMI, subcutaneous fat volume, and visceral fat volume.

To further contextualize our findings, it is important to consider the types of fat depots, rather than the weight or fat content. BMI has traditionally been used as a tool for assessing obesity and metabolic risk; however, it has certain limitations. While BMI is a straightforward and convenient metric that allows for the rapid classification of individuals into categories of normal weight, overweight, or obese, it fails to distinguish fat from lean muscle mass and reflect body fat distribution (16). Individuals with a normal or even low BMI may still experience significant metabolic risks due to increased levels of VAT. Substantial studies have acknowledged that excess visceral adiposity poses a more significant threat to abdominal obesity, metabolic disorders, diabetes, and cardiovascular diseases (17, 18). Such individuals are often not recognized as high-risk populations in standard BMI assessments, potentially delaying timely intervention and treatment (19). This phenomenon is particularly pronounced in Asian populations (20). Due to genetic predispositions, lifestyle factors, and dietary habits, these individuals may have elevated VAT despite a BMI within the normal range. This characteristic places this subgroup at a higher metabolic risk, including conditions such as insulin resistance, diabetes, hypertension, and cardiovascular diseases (21). Therefore, reliance solely on BMI as a standard assessment tool may underestimate the metabolic health risks faced by specific populations. WC offers a convenient method to evaluate body fat distribution and has been demonstrated a stronger correlation with the absolute amount of VAT compared to BMI (22). Consequently, WC is incorporated into the diagnostic criteria for MetS as an indicator of abdominal fat. It is recommended that overweight or obese individuals with a potential metabolic risk undergo WC evaluations to assess abdominal obesity (23). Even for those with lower BMI categories, WC measurements are still significant as those with low BMI but elevated WC face a considerably higher risk of morbidity and mortality (24). However, WC also has certain limitations in clinical application. WC measurements can be influenced by various factors, including methodology, individual factors (such as meal, breathing and posture), as well as ethnicity and gender, leading to considerable variability in results (25, 26). Furthermore, WC primarily reflects the overall amount of abdominal fat without distinguishing between different types. Therefore, WC may not provide sufficient precision in assessing metabolic risk, particularly in individuals with normal or low BMI.

In this regard, PrFV, as a direct measure of visceral fat, might offer greater specificity and sensitivity. The positive correlation between PrFV and metabolic profiles and MetS showed in our study suggest that PrFV may accurately reflect the metabolic activity and risk. The non-invasive nature of PrFV measurement, performed by trained professionals and multidimensional quantification, position PrFV as a promising complementary or even superior clinical marker to WC in assessing the risk of MetS. In our study, we observed a superior performance of PrFV in predicting MetS than SFV and VFV. The optimal cutoff value of PrFV was 62.5 cm^3^ and the AUC was 0.753, with a sensitivity of 62.3% and a specificity of 82.1%. Several studies reported the predictive value of perirenal fat measured by MRI in metabolic diseases. Wang et al. employed MRI to quantify perirenal fat thickness (PrFT) and investigated the association between PrFT and MetS in adults with overweight and obesity. The optimal cut-off value of PrFT was 9.15 mm, with a sensitivity of 0.683 and specificity of 0.549 (AUC = 0.610). Likewise, Dong et al. utilized MRI (including MRI fat fraction and R2* mapping) to quantify renal sinus fat in patients with type 2 diabetes mellitus (T2DM) (27). They found that renal sinus fat dysfunction was independently associated with T2DM and the AUC of combining fat fraction and R2* mapping for predicting T2DM was 0.729, with a sensitivity of 0.632 and specificity of 0.804 (28). Hence, considering the non-invasive and convenient nature of ultrasonography, along with its comparable predictive performance for metabolic diseases to MRI, the use of ultrasonography to measure PrFV holds significant value for the diagnosis and management of MetS. We then proposed a preliminary flowchart for clinical use of PrFV in assessing MetS (**Supplementary Figure S1**). Notably, while our study demonstrates a significant association between increased PrFV and MetS risk, the current cross-sectional design and sample size limit our ability to definitively establish a validated, generalizable PrFV cutoff value. The suggested value of 62.5 cm³ was not a pre-defined threshold in our analysis, but rather an illustrative value. Future research should prioritize large, prospective cohort studies to establish clinically relevant PrFV thresholds, integrating PrFV measurements with other clinical risk factors and validating these thresholds among different races and regions to enhance predictive accuracy and clinical applicability.

As we explore the methodologies for assessing PRAT, it becomes evident that different imaging techniques offer unique advantages. Currently, several methodologies exist for assessing PRAT, including ultrasonography, CT, MRI, and Positron Emission Tomography (PET). MRI and PET, however, are less frequently applied due to complexity and ionizing radiation, except under specific circumstances such as differentiating perirenal lesions or measuring metabolic parameters (29, 30). Ultrasonography and CT, on the other hand, are widely employed in clinical studies, each offering distinct advantages. Ultrasonography is convenient, rapid, and radiation-free, while CT provides comprehensive and high-resolution imaging (31). Ultrasound enabled clinicians to use a non-invasive and repeatable method to quantify the PRAT amount with approximate accuracy to CT. In our study, we innovatively utilized ultrasonography to measure PRAT thickness from three dimensions and, therefore, were able to calculate the PrFV and better quantify PRAT (32). Furthermore, in order to eliminate the influence of fat deposition on the correlation results and to compare the predictive value of perirenal fat, SAT, and VAT in the presence of MetS, we utilized MRI-based algorithms combined with artificial intelligence to segment abdominal fat at different layers, thereby obtaining the volumes of SAT and VAT (SFV and VFV).

Accumulating epidemiological evidence underscores the link between perirenal fat and hypertension. Hypertension is one of the most common manifestations of MetS. PrFV may play a pivotal role in the development and progression of hypertension. Ricci et al. reported that PrFT was significantly increased in the hypertensive group (13.6 ± 4.8 vs. 11.6 ± 4.1 mm) and that PrFT could independently predict SBP in the morbidly obese population (R^2^ = 0.129, β = 0.160, *P* = 0.022). After a follow-up for 10–12 months post sleeve-gastrectomy, a reduction of perirenal fat thickness was observed alongside the decrease in anthropometric parameters, blood pressure, and serum lipid level (33). Several cross-section studies also reported a positive correlation of PrFT and 24-hour mean DBP (r = 0.34) (34), as well as office blood pressure (SBP, r = 0.213; DBP, r = 0.215) (35). However, current evidence is primarily based on studies involving morbidly obese patients, and the general impact of perirenal fat on blood pressure requires further confirmation, particularly in the general or healthy-obese populations. Possible mechanisms underlying the association between PRAT and hypertension involves multiple complex physiological and pathological mechanisms, primarily including neuroregulatory mechanisms, endocrine function and inflammatory responses, and physical compression. The neurogenic regulation is often referred to as the adipose afferent reflex. The afferent nerve within PRAT could convert chemical sensory signals into neural impulses and project to dorsal root ganglia and hypothalamic paraventricular nucleus, thereby regulating sympathetic nerve activities (36). Li et al. identified a critical intersection joint of this neuronal circuit, calcitonin gene-related peptide (CGRP), as an endogenous counteractor of pathological high blood pressure. Chronic, pro-hypertensive factors stimulate PRAT afferent nerve and suppress the synthesis of CGRP. Ablation of PRAT could reverse pathological hypertension by restoring CGRP synthesis and its blood pressure-neutralizing capabilities (9). Additionally, adipokines and cytokines released by PRAT (such as leptin and adiponectin) may have an adjacent effect on renal function and blood pressure, participating in the onset of hypertension via humoral regulation. In hypertensive patients, the increased synthesis of leptin in PRAT leads to elevated local leptin concentrations in the kidneys, which in turn promotes the proliferation of glomerular endothelial cells through the MAPK signaling pathway and regulates the renin-angiotensin-aldosterone system, ultimately resulting in increased blood pressure (37). Inflammatory factors secreted by PRAT, such as TNF-α and IL-1β, also play a significant role in the pathogenesis of hypertension. These inflammatory factors affect normal kidney function by activating the sympathetic nervous system, impairing renal vascular endothelial function, and inducing renal fibrosis, ultimately leading to increased blood pressure (38). Excessive physical fat compression may also activate renal sympathetic nerve activity and renin-angiotensin-aldosterone system (39).

Our findings align with existing literature regarding the association between PRAT and various metabolic risk factors. A Chinese cross-sectional study involving 867 subjects with diabetes reported that PrFT tertiles were increasingly correlated with TG, TC, and insulin resistance, while negatively correlated with HDL-C. PrFT was independently associated with the occurrence of metabolic dysfunction-associated fatty liver disease (40). Similar results were also observed in an Italian overweight and obese cohort by Carlo et al. (41), where PrFT had a direct correlation with higher BMI, WC, TG, homeostatic model assessment of insulin resistance (HOMA-IR), and lower HDL-C, independent of other anthropometric and hemodynamic parameters. Carlo et al. speculated that excessive accumulation of PRAT may secret adipokine and cytokines that promote pro-inflammatory macrophages, trigger inflammation and worsen insulin resistance. Guillem et al. investigated the correlation between different abdominal fat layers (perirenal fat, preperitoneal fat, omental fat, and subcutaneous fat) and MetS features in patients with obesity (42). Patients meeting the ATP III criteria for MetS exhibited thicker perirenal (both left and right) and omental fat depots. Both perirenal fat and omental fat could independently predict later MetS onset (cut-off point of 22.5 mm in males/12.5 mm in females for perirenal fat; 54 mm in males/34 mm in females for omental fat). In particular, omental fat, a major component of visceral fat, showed strong correlations with metabolic risk factors such as serum glucose levels (r = 0.22; *P* < 0.05) and HOMA-IR (r = 0.43; *P* < 0.001). No statistical differences were found between fat depots in the group of hypertension and dyslipidemia, except for a negative correlation between HDL level and omental fat thickness (r = −0.22; *P* < 0.05) as well as left perirenal fat (r = −0.23; *P* < 0.0001). On the contrary, subcutaneous fat layers were thicker in women and negatively correlated with omental fat, suggesting a protective role against metabolic risks. Moreover, Guillem’s team recently conducted an open-label, single-centre, randomized, controlled study (EudraCT: 2019-000979-16) to investigate the influence of antidiabetic drugs on different abdominal fat layers (43). A significant reduction of perirenal fat beyond other fat layers was observed, either treated with metformin alone or combined with dapagliflozin. Specifically, the loss of perirenal fat significantly correlated with HOMA-IR (r = 0.74, *P* = 0.017) and improved insulin performance, which is a core path in metabolic regulation and a recognized mechanisms underlying dapagliflozin. In summary, these findings indicate that excessive visceral fat and perirenal fat were closely related to increased matebolic risks. Perirenal fat may not only accompany but also antedates and participate in the pathogenesis of MetS. PRAT exhibits promising potential as a therapeutic target beyond its role as a mere surrogate marker.

An intriguing discovery from our study reveals an independent and positive correlation between PrFV and hyperuricemia in supplementary analysis. Although not explicitly listed in the diagnostic criteria of MetS, hyperuricemia is a common metabolic disease manifested as excessive accumulation of uric acid in plasma. It is well known that hyperuricemia is linked to gout, diabetes, and cardiovascular diseases (44). Yang et al. reported that both perirenal and pararenal fat thickness positively correlated with SUA level, particularly among males and healthy patients (45). Comparable findings have also been observed in populations with hyperlipemia (32), chronic kidney disease (46), and type 2 diabetes mellitus (40). The robust association between these factors may be explained by several potential mechanisms. First, excessive fat accumulation and its physical compression to medullary vasa recta and tubules may decrease blood flow velocity, thereby enhancing the reabsorption of uric acid (47). Second, excess adipose tissue may directly increase the secretion of uric acid via downregulating adiponectin production and augmenting oxidative stress and chronic inflammation (48, 49). Moreover, multiple studies have demonstrated a close association between PRAT accumulation and renal function decline in obese, gout and diabetic patients, manifested by decreased glomerular filtration rate, reduced effective renal plasma flow, increased renal vascular resistance, and elevated microalbuminuria (50, 51). The mechanisms by which PRAT cause gout and diabetic nephropathy primarily involve mechanical fat-induced compression and endocrine dysfunction. Excessive accumulation of PRAT may exert mechanical pressure on the kidneys, impacting the renal vasculature and renal parenchyma. This can lead to increased renal vascular resistance and interstitial hydrostatic pressure, resulting in decreased renal blood flow and glomerular filtration rate, thereby impairing glomerular filtration function (52). Chen et al. reported a positive correlation between PRAT thickness and both renal vascular resistance and afferent arteriolar resistance, indicating that PRAT may influence renal function by altering renal hemodynamic parameters (53). Additionally, PRAT regulates the metabolic state and inflammatory response of the kidneys by secreting various bioactive substances, such as adipokines and pro-inflammatory cytokines. These bioactive substances may drive oxidative stress, disrupt the homeostasis of the renal microenvironment, and cause damage to the renal tubules and glomeruli, which further exacerbates nephropathy (54, 55).

Considering that PRAT plays a significant role in metabolic diseases, exploring and implementing diverse intervention strategies targeting PRAT holds the potential to revolutionize the management of related metabolic disorders. Lifestyle modifications are fundamental measures in the management of hypertension and other metabolic diseases. By optimizing dietary patterns, increasing physical activity, and promoting weight loss, the accumulation of PRAT can be effectively curtailed. Implementing a diet rich in fish oil (56), adjusting meal frequency to two meals per day (57), and engaging in high-intensity interval training (58) have all been shown to reduce PRAT and improve metabolic status. Certain medications aimed at controlling cardiovascular disease risk factors may also have a positive impact on PRAT. Administration of irisin has been shown to significantly reduce body weight, fat mass, and free fatty acid levels in high-fat diet-fed mice, while increasing the expression of PRAT-related functional proteins such as uncoupling protein-1 and heme-oxygenase-1 (59). Additionally, the combination of dapagliflozin and metformin treatment significantly reduced PRAT layers in obese patients with type 2 diabetes, accompanied by reductions in plasma leptin, C-reactive protein, and urinary microalbumin levels (43). Physical therapy such as extracorporeal shock wave therapy (ESWT) has been reported to treat cellulite and have an improving effect on abdominal lipolysis and lipid metabolism (60, 61). Focused power ultrasound, as an emerging non-invasive treatment, is gradually being explored for its potential application in the modification of PRAT. This technique achieves localized heating by precisely focusing ultrasound waves on the PRAT region, elevating tissue temperature to approximately 50-55°C (an increase of 13-18°C above baseline) within a short duration. This process induces degeneration of adipose tissue rather than ablation, thereby achieving the goal of modification therapy. An exploratory study conducted on a small Chinese population indicated that focused ultrasound treatment for PRAT demonstrated good safety and significant antihypertensive effects in both office blood pressure and ambulatory blood pressure monitoring (62). In light of this, several ongoing multicenter randomized controlled trials (e.g., NCT06018493 and NCT06283758 for primary hypertension, as well as NCT06225723 for metabolic associated fatty liver disease) will further evaluate the efficacy and safety of this modification approach directly targeting PRAT in metabolic diseases. Last but not least, emerging therapies may reduce the risk of metabolic diseases by modulating the cellular composition and function of PRAT, thereby optimizing its metabolic characteristics. For instance, cold exposure and β3-adrenergic receptor stimulation may provide new therapeutic avenues for metabolic diseases by influencing the energy metabolism pathways of PRAT (49, 63). With advancements in regenerative medicine and gene therapy, future strategies targeting PRAT may include cell and gene therapies.

Our study has several strengths and limitations. As a sub-analysis of a multicenter, randomized clinical study, our research benefited from rigorous supervision and monitoring, ensuring the reliability and credibility of the data. We utilized an innovative sonographic method to assess PRAT volume and employed MRI-based algorithms to precisely differentiate and quantify subcutaneous and visceral fat. After adjustment for age, gender, smoking status, drinking status, BMI and abdominal fat volume, a robust and positive association still exists between PRAT and the incidence of MetS. Our finding provided new insight into PRAT, suggesting PRAT a surrogate marker and a potential therapeutic target for MetS. Given the non-invasive nature of ultrasonography for measuring PrFV, our study presents a practical tool for clinicians to assess visceral fat accumulation and implement early interventions aimed at reducing metabolic risks. We propose a preliminary flowchart for clinical use of measuring PrFV in MetS management, hoping to underscore the importance of monitoring perirenal fat as part of routine clinical evaluations to improve metabolic health outcomes. However, there were certain limitations in this study. Primarily, due to the small sample size and the cross-sectional design, we are unable to establish a causal relationship between PRAT and MetS. Furthermore, the study population was selected from a clinical trial focusing on invasive therapy for hypertension, which inherently introduces recall bias and selection bias. Moreover, as we did not collect dietary information or measure insulin resistance, unknown confounders may interfere with the result even if we have adjusted for common confounders. Therefore, additional clinical studies are necessary to further explore the significance of PRAT in the context of MetS.

## Data Availability

The original contributions presented in the study are included in the article/**Supplementary Material**. Further inquiries can be directed to the corresponding author.

## Supplementary material

Supplementary Figure 1 The flowchart for clinical use of PrFV in assessing MetS. MetS, metabolic syndrome; WC, waist circumstance; PrFV, perirenal fat volume.
