# Supplementary material for: Perirenal fat as a potential marker and therapeutic target for metabolic syndrome: insights from a multicenter randomized controlled trial
Source: Front Endocrinol (Lausanne). 2025 May 23;16:1557701. doi: 10.3389/fendo.2025.1557701 (PMC12140993; doi:10.3389/fendo.2025.1557701)
Supplement: Supplementary Figure 1 — The flowchart for clinical use of PrFV in assessing MetS. MetS, metabolic syndrome; WC, waist circumstance; PrFV, perirenal fat volume. [file Table1.docx]

Supplementary Table 1．Logistic regression models between PrFV and metabolic disorders

|  | **Crude model (Model 1)** | | **Model 2** | | **Model 3** | |
| --- | --- | --- | --- | --- | --- | --- |
|  | Odds ratio  (95%CI) | P-value | Odds ratio (95%CI) | P-value | Odds ratio  (95%CI) | P-value |
| **Hyperlipidemia** |  |  |  |  |  |  |
| Average volume | 1.02 (1.01-1.04) | 0.01 | 1.02 (1.01-1.04) | 0.01 | 1.02 (1.00-1.04) | 0.04 |
| Q1 | Ref. |  | Ref. |  | Ref. |  |
| Q2 | 2.1 (0.79-5.75) | 0.14 | 1.84 (0.66-5.22) | 0.25 | 2.20 (0.69-7.15) | 0.18 |
| Q3 | 4.86 (1.77-14.36) | 0.003 | 3.74 (1.23-11.43) | 0.02 | 2.99 (0.90-10.57) | 0.08 |
| **Hyperglycemia** |  |  |  |  |  |  |
| Average volume | 1.01 (0.99-1.02) | 0.47 | 1.01 (0.99-1.02) | 0.32 | 1.00 (0.99-1.02) | 0.64 |
| Q1 | Ref. |  | Ref. |  | Ref. |  |
| Q2 | 1.19 (0.37-3.86) | 0.77 | 1.29 (0.40-4.30) | 0.68 | 0.85 (0.21-3.38) | 0.82 |
| Q3 | 2.03 (0.69-6.29) | 0.20 | 2.44 (0.79-8.2) | 0.13 | 1.80 (0.47-7.40) | 0.40 |
| **Hyperuricemia** |  |  |  |  |  |  |
| Average volume | 1.03 (1.01-1.04) | <0.001 | 1.03 (1.01-1.04) | 0.001 | 1.02 (1.01-1.04) | 0.02 |
| Q1 | Ref. |  | Ref. |  | Ref. |  |
| Q2 | 1.44 (0.44-4.931) | 0.55 | 1.37 (0.41-4.82) | 0.61 | 1.09 (0.28-4.31) | 0.90 |
| Q3 | 9.41 (3.17-31.68) | <0.001 | 8.22 (2.66-28.7) | <0.001 | 5.20 (1.47-20.45) | 0.01 |

PrFV, perirenal fat volume; CI Confidence interval

Model 1 was adjusted for none. Model 2 was adjusted for age and gender. Model 3 was further adjusted for smoker, drinker, BMI, subcutaneous fat volume and visceral fat volume.
