# Supplementary figures and images for: Perirenal fat as a potential marker and therapeutic target for metabolic syndrome: insights from a multicenter randomized controlled trial
Source: Front Endocrinol (Lausanne). 2025 May 23;16:1557701. doi: 10.3389/fendo.2025.1557701 (PMC12140993; doi:10.3389/fendo.2025.1557701)

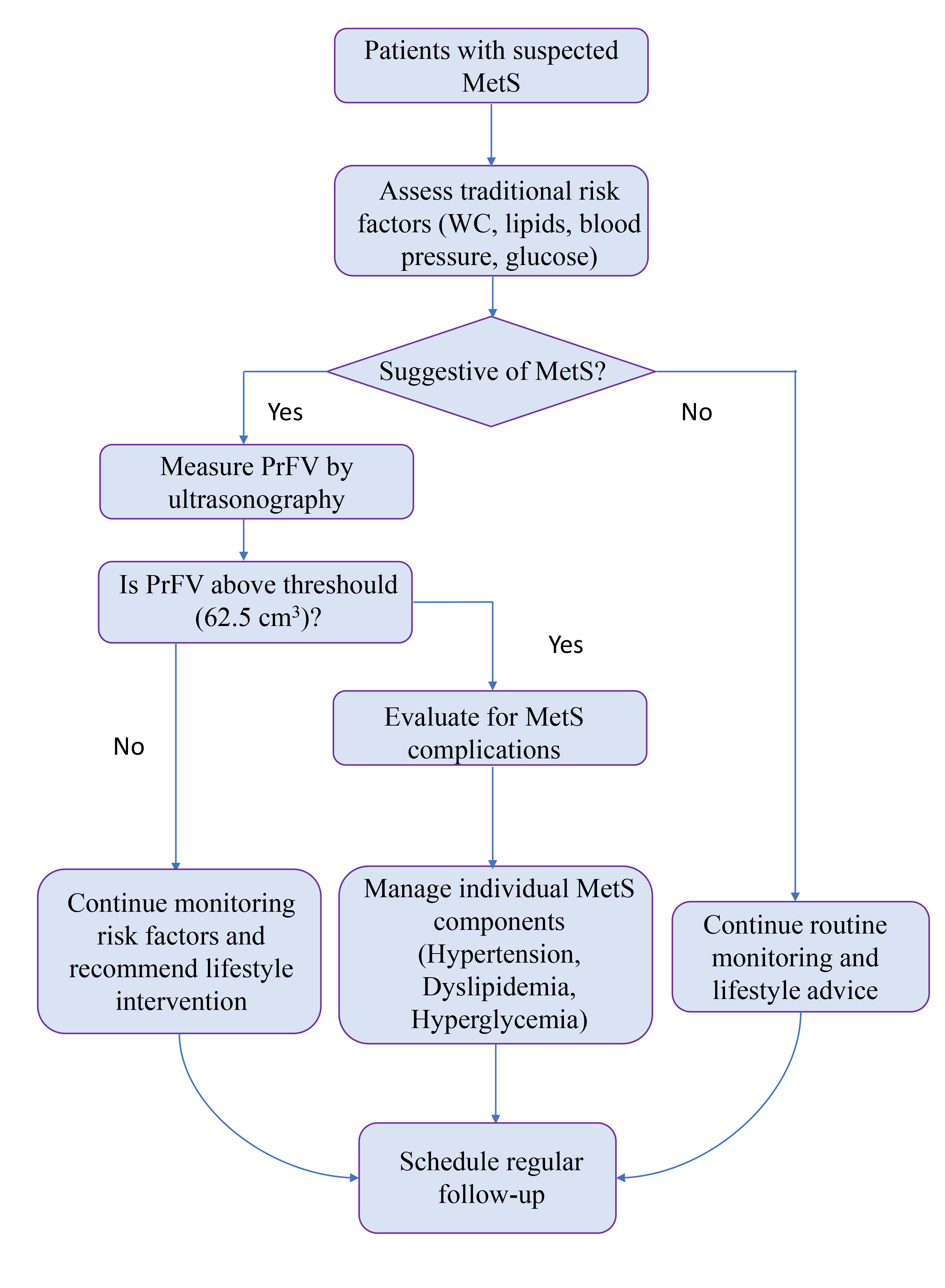

Supplement: Supplementary file 2 [file Image1.tif]
